# Regulation of Hedgehog Signaling by Myc-Interacting Zinc Finger Protein 1, Miz1

**DOI:** 10.1371/journal.pone.0063353

**Published:** 2013-05-03

**Authors:** Jiuyi Lu, Minyong Chen, Xiu-Rong Ren, Jiangbo Wang, H. Kim Lyerly, Larry Barak, Wei Chen

**Affiliations:** 1 Department of Medicine, Duke University, Durham, North Carolina, United States of America; 2 Department of Surgery, Duke University, Durham, North Carolina, United States of America; 3 Department of Cell Biology, Duke University, Durham, North Carolina, United States of America; Indiana University School of Medicine, United States of America

## Abstract

Smoothened (Smo) mediated Hedgehog (Hh) signaling plays an essential role in regulating embryonic development and postnatal tissue homeostasis. Aberrant activation of the Hh pathway contributes to the formation and progression of various cancers. In vertebrates, however, key regulatory mechanisms responsible for transducing signals from Smo to the nucleus remain to be delineated. Here, we report the identification of Myc-interacting Zinc finger protein 1 (Miz1) as a Smo and Gli2 binding protein that positively regulates Hh signaling. Overexpression of Miz1 increases Gli luciferase reporter activity, whereas knockdown of endogenous Miz1 has the opposite effect. Activation of Smo induces translocation of Miz1 to the primary cilia together with Smo and Gli2. Furthermore, Miz1 is localized to the nucleus upon Hh activation in a Smo-dependent manner, and loss of Miz1 prevents the nuclear translocation of Gli2. More importantly, silencing Miz1 expression inhibits cell proliferation *in vitro* and the growth of Hh-driven medulloblastoma tumors allografted in SCID mice. Taken together, these results identify Miz1 as a novel regulator in the Hh pathway that plays an important role in mediating Smo-dependent oncogenic signaling.

## Introduction

Hedgehog (Hh) signaling is an evolutionarily conserved pathway that plays a crucial role in tissue development and homeostasis in both invertebrates and vertebrates [1]. Hh proteins are morphogens secreted by specialized Hh-producing cells. Sonic hedgehog (Shh) is the most studied Hh ligand in vertebrates [1]. In the absence of Hh protein, Patched1 (Ptch1), a twelve-transmembrane Hh protein receptor on the plasma membrane, catalytically inhibits the seven transmembrane receptor Smoothened (Smo) receptor, the choke-point of downstream signaling [2]. In the presence of Hh, Ptch1 loses its ability to inhibit Smo receptors, allowing Smo to become active. As a consequence, Gli transcription factors translocate into the nucleus and activate target gene expression. The importance of Hh regulation to normal development is exemplified by the occurrence of developmental disorders as a result of germline malfunctioning of the pathway. Additionally, aberrant activation of Hh signaling has been linked to many forms of human cancers, such as basal cell carcinoma (BCC) and medulloblastoma [3], [4], [5], [6].

The primary cilium is an organelle that protrudes from the surface of most vertebrate cells [7]. It has been shown that proteins involved in cilium formation, including Kif3a, IFT88 and IFT172, are also required for Hh signaling [8], [9]. In addition, most of the key components in the Hh pathway, such as Smo, Gli, and Sufu, are found localized in primary cilia upon Hh activation [10], [11], [12], [13], [14], [15]. Specifically, in the absence of Shh, Ptch1 resides at the base of primary cilia and precludes Smo from associating with the cilium [11], [16]. Upon activation, Ptch1 and its ligand Shh move out of the cilium and become internalized into the cytoplasm [16]. Subsequently, Smo translocates into the cilium [11] and allows the production of an activated Gli2 concentrated at the distal ends of the primary cilium in a Smo-dependent fashion [12], [13]. The translocation of Gli2 to cilia occurs within minutes of ligand stimulation [12], however, it remains unknown how activated Gli2 is moved into the nucleus in order to control gene transcription downstream of Hh activation.

Miz1 is a member of the POZ domain/zinc finger transcription factor family that contains a BTB/POZ domain at its N-terminus followed by 13 zinc finger domains [17]. Miz1 is expressed ubiquitously during development and can function as either a transcription activator or repressor depending on its binding partners [18]. Miz1 was first identified as a Myc-interacting protein. The C-terminus of Miz1 has been found to bind and recruit Myc oncoprotein to core promoter elements of targeting genes to overcome Miz1-mediated growth arrest effect [17]. In addition, Miz1 induces cell cycle arrest by activating the transcription of p15INK4b and p21CIP1 [19], [20], [21]. Both transcriptional activation and repression activity of Miz1 require the intact POZ domain [17], [19]. Furthermore, Miz1 is required for early embryonic development during gastrulation as *Miz1^−/−^* embryos do not survive beyond E6.5 day [22]. Despite its critical involvement in embryogenesis, the underlying molecular mechanism of Miz1-dependent regulation of normal development has yet to be discovered.

In the present study, we report that Miz1 plays an important role in regulating Hh signaling. Miz1 binds to Smo and Gli2, accumulates in primary cilia, and translocates into the nucleus in a Smo-dependent manner. Knockdown of endogenous Miz1 suppresses cell proliferation *in vitro* and tumorigenesis of a Hh-driven medulloblastoma cell line PZp53^MED1^ in SCID mice. These data suggest that Miz1 is required for orchestrating normal Hh signaling and it functions as a potential oncogene in promoting cell proliferation in Hh-dependent tumors.

## Materials and Methods

### DNA constructs

Both Myc-Miz1 and Flag-Miz1 expression constructs were kindly provided by Dr. Martin Eilers (University of Würzburg, Germany). Mutant Miz1 constructs including Myc-Miz1ΔPOZ, Flag-Miz1ΔPOZ, and Flag-Δ641–714 were generated by mutagenesis and verified by DNA sequencing. The HA-Gli2 construct was provided by Dr. Philip Beachy (Stanford University). The Gli-luciferase reporter was a gift from Dr. Frederic de Sauvage (Genentech Inc.). The pRL-TK Renilla luciferase construct was purchased from Promega.

### Cells and transfection

NIH 3T3 cells were cultured in DMEM supplemented with 10% bovine calf serum. The shRNA for the mouse Miz1 gene was constructed in pLKO.1-puro vector and purchased from Sigma-Aldrich. Miz1 knockdown cells were generated using lentivirus-mediated delivery of shRNA. Stable knockdown lines were cultured in DMEM supplemented with 10% bovine calf serum and 1 μg/μl puromycin. Smo knockout (Smo*^−/−^*) MEF cells [23] were generously provided by Dr. James Chen (Stanford University) and cultured in DMEM supplemented with 10% fetal bovine serum (FBS). C3H10T1/2 cells were grown in BME supplemented with 10% bovine calf serum, 10 ml sodium bicarbonate, and 2 mM L-glutamate. HEK 293 cells were culture in MEM supplemented with 10% FBS and 1% penicillin/streptomycin. PZp53^MED1^ cells [24] were a kind gift from Dr. Matthew Scott (Stanford University) and cultured in DMEM supplemented with 10% FBS. Transient transfection of all cell types was carried out using Fugene 6 (Promega) unless otherwise stated.

### Yeast two-hybrid screening

The DNA sequence encoding the last 249 amino acids in the Smo C-terminus was cloned into pGBT-10 yeast expression vector (Clontech). This pGBT-10-Smo-C bait construct was transformed into Y190 yeast strain together with a human brain cDNA library (Clontech). Library plasmids carried by the positive clones were rescued and sequenced. The interaction specificity was further confirmed by co-transforming the isolated plasmid with either pGBT-10-Smo-C or pGBT-10 vector into yeast and selected on -His/-Ade plates.

### Pharmacological regulation of Hh signaling

To activate or inhibit Smo, NIH 3T3 or C3H10T1/2 cells were treated with one of the following agents alone or in combination, Smo agonist SAG (0.25 μM), cyclopamine (3 μM), or GDC-0449 (1 μM). All compounds were dissolved in sterile DMSO, and DMSO was used in control treatments.

### Hh-responsive luciferase reporter assay

The Hh-responsive luciferase reporter assay was performed in NIH3T3, *Smo^−/−^* MEF, or C3H10T1/2 cell lines. Briefly, cells were transfected with the Gli reporter plasmid (9× Gli-binding sites coupled to the firefly luciferase) with a *Renilla* luciferase control pRL-TK plasmid (Promega), and luciferase activities were measured using a Dual-Luciferase Reporter Assay System (Promega). The activity of the Gli reporter was defined as the ratio of firefly/*Renilla* luciferase activities. Each experimental point was performed in quadruplicate, and 2–3 independent experiments were included in final analyses.

### Two-step Real-Time PCR

Two-step real-time PCR was performed to compare the expression of Hh pathway components in NIH3T3 cells or PZp53^MED1^ cells. Total RNA was extracted from cells using Trizol (Invitrogen, Carlsbad, CA). Equal amounts of RNA were used as templates for all reactions. Total cDNA was produced using a SuperScript First-Strand Synthesis Kit (Invitrogen, Carlsbad, CA). Real-time PCR reactions were carried out using 50 ng of cDNA as the template and amplified using SYBR Green Master Mix (Applied Biosystems) and specific oligonucleotide primers for target genes. The *18S* gene was used as the internal control.

### Immunocytochemistry

Cells seeded in glass-bottom dishes were treated with DMSO or 0.25 μM SAG as indicated. To detect the location of endogenous Miz1, cells were fixed in 4% paraformaldehyde at room temperature for 10 min and permeabilized using 0.2% Triton X-100 in PBS. After blocking with 5% BSA in PBS, cells were stained with anti-Miz1 (1∶100, a gift from Dr. Robert Tjian) and anti-acetylated tubulin mAb (1∶1000, Sigma) overnight at 4°C. For Gli2 studies, cells were fixed in cold methanol for 5 min and blocked with 5% BSA in PBS. Cells then were stained with anti-Gli2 (1∶1000, a gift from Dr. Suzie J. Scales) and anti-detyrosinated tubulin Ab (1∶1000, Millipore). The Alexa 488- and Alexa 561-conjugated secondary antibodies (Invitrogen) were used subsequently. The nuclei of the cells were stained with DAPI-containing mounting medium (Vector Laboratories, Inc. Burlingame, CA). Confocal images were obtained with a 3I spinning disk confocal microscope equipped with an Electron Multiplying-Charge Coupled Device (EM-CCD) camera and a Z galvanometer stage. For the quantification of signal intensity in cilia, each primary cilium revealed by acetylated/detyrosinated tubulin staining was outlined manually. The fluorescent intensity of Miz1 or Gli2 within the outlined areas in cilia was measured using Metamorph software (Universal Imaging Corporation), and approximately 100–300 cilia were analyzed. For background correction, the signal intensity of a nearby unstained region with the same area as the outlined region was subtracted from the ciliary fluorescence of Miz1 or Gli2.

### Cell proliferation

Cells were seeded in 96-well plates and allowed to grow in serum-free medium for overnight. A WST-1 assay was performed by adding 10 μl of WST-1 reagent directly into the culture medium and incubating for 2 h at 37°C. Plates were then read by a spectrophotometer to measure the absorbance at 450 nm and a reference wavelength of 630 nm. The ^3^H-thymidine incorporation experiment was conducted as previously described [25].

### Cell fractionation

Cells were harvested and washed twice with ice-cold PBS followed by resuspending in buffer A (10 mM HEPES pH 7.9, 1.5 mM MgCl_2_, 10 mM KCl, 0.5 mM DTT) plus protease inhibitor cocktail (1 mM PMSF, 10 μg/ml aprotinin, 10 μg/ml leupeptin, 10 μg/ml pepstatin A). Cells were tumbled for 30 min at 4°C and then centrifuged for 5 min at 16,000 g. The resulting supernatant is defined as the cytoplasmic extract. The pellet was resuspended in buffer B (20 mM HEPES pH 7.9, 420 mM NaCl, 1.5 mM MgCl_2_, 0.2 mM EDTA, 25% glycerol, 0.5 mM DTT) with the above protease inhibitors and defined as the nuclear fraction.

### Tumorigenesis of medulloblastoma in vivo

PZp53^MED1^ cells infected with lentivirus encoding scrambled shRNA or shRNAs against Miz1 were selected with 1 μg/ml puromycin. The resulting stable scrambled (Scr) control and Miz1 knockdown cells were used in in vivo tumorigenesis experiments. For subcutaneous inoculation, 5×10^4^ of either Scr control or Miz1 knockdown (#3 and #5) cells in 0.05 ml of Hank's Balance Salt Solution were mixed with Matrigel (1∶1) and injected into SCID mice. Five mice were injected in each group. Tumor size was measured with a caliper and the tumor volume was defined as [length × width ×0.5× (length + width)]. All of the tumors were harvested and weighed when the largest tumor reached 2000 mm^3^. All animal procedures were performed using protocols approved by the Duke University Animal Care and Use Committee (protocol number A311-12-12).

## Results

### Miz1 positively regulates Hh signaling by interacting with Smo and Gli2

**Figure 1:**
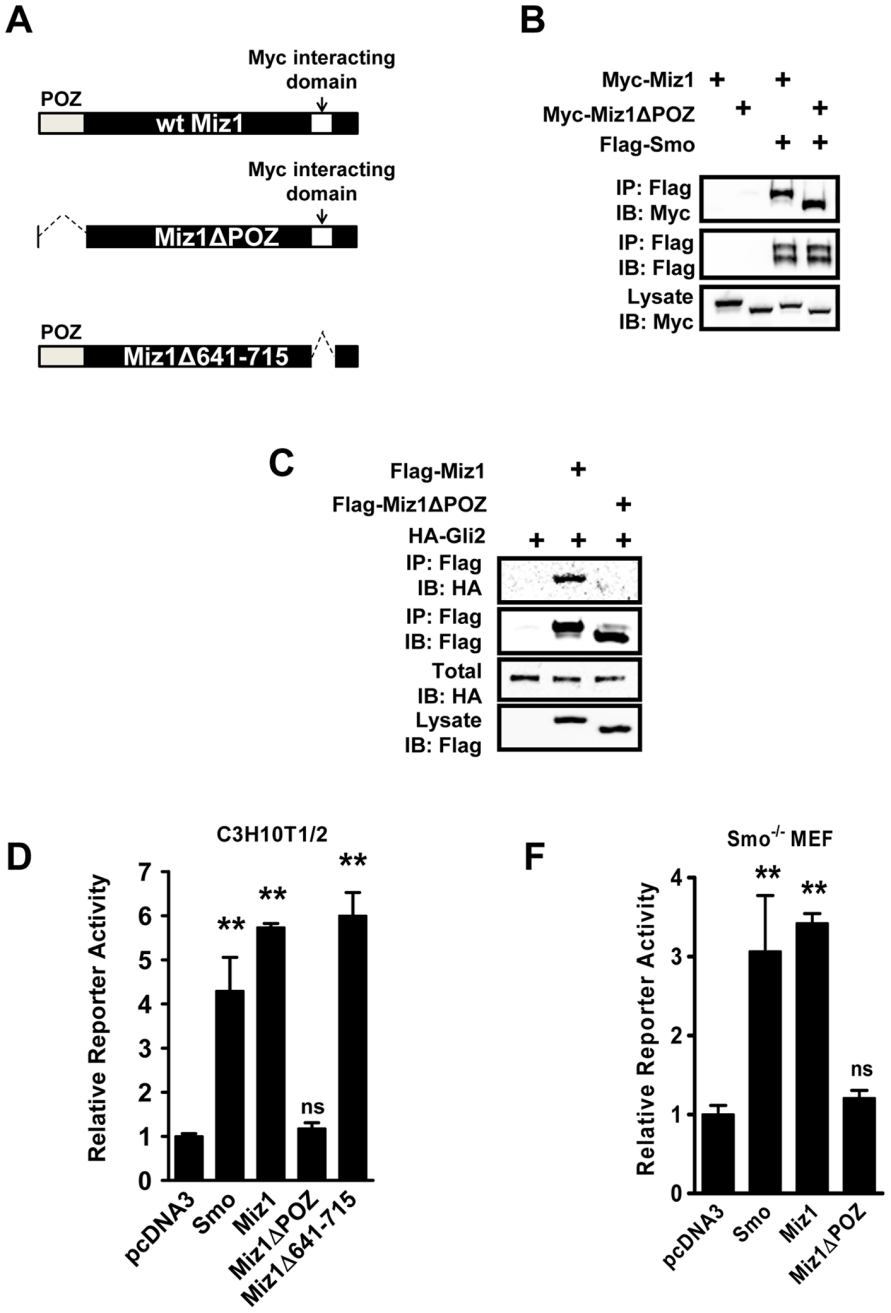
Identification of Miz1 as a positive regulator of Hh signaling. (A) Illustration of Miz1 mutants. Wildtype (wt) Miz1 (total 803 amino acid residues) contains a POZ domain (amino acid residue 1–104) and a Myc-interacting domain (amino acid residue 641–715) POZ domain is deleted in Miz1ΔPOZ mutant, and the Myc-interacting domain is deleted in Miz1Δ641–715 mutant. (B) Miz1 interacts with Smo, and such interaction is independent of the POZ domain of Miz1. HEK293 cells were transiently transfected with Myc-tagged Miz1 or a POZ domain deletion mutant Miz1ΔPOZ along with Flag-Smo as indicated. Co-immunoprecipitation experiments were performed using the Flag antibody. The presence of Miz1 full length and Miz1ΔPOZ was detected using the Myc antibody. (C) Miz1 also interacts with Gli2, and the POZ domain of Miz1 is required for the interaction between Miz1 and Gli2. HEK293 cells were transiently transfected with HA-Gli2 together with vector, Flag-Miz1, or Flag- Miz1ΔPOZ as indicated. Co-immunoprecipitation experiments were performed using the Flag antibody. The presence of Gli2 was detected using the HA antibody. (D) Miz1 promotes the activation of Gli activity. C3H10T1/2 cells were transfected with the Hh-responsive reporter (9× Gli-luciferase) together with one of the corresponding constructs as indicated. The Gli reporter activity was determined using the ratio of firefly/*Renilla* luciferase light-units. The value in cells transfected with pcDNA3 was normalized to 1 and used as control, and the relative Gli activity was obtained by comparing to the control. Data shown in the graph represent the mean ± SEM (n = 3, **: *p*<0.001, ns: not significant, compared to the pcDNA3 sample, t test). (F) Miz1 activates Gli activity downstream of Smo receptor. *Smo^−/−^* MEF cells were transfected with the Hh-responsive reporter (9×Gli-luciferase) and pRL-TK together with one of the corresponding constructs as indicated. Gli reporter activities were determined as described in (D). Data shown in the graph represent the mean ± SEM (n = 3, **: *p*<0.001, ns: not significant, compared to the pcDNA3 sample, t test).

The C-terminus of *Drosophila* Smo receptor contains multiple phosphorylation sites, and the phosphorylation of the C-terminus has been implicated in controlling the activation of Hh signaling in invertebrates [3], [26]. However, the C-terminus of Smo diverges significantly in vertebrates suggesting that vertebrate Smo receptors are regulated differently [26]. In order to identify potential regulators of Hh signaling that interact with mammalian Smo, we performed a yeast-two-hybrid screen against a human brain cDNA library using the last 249 amino acids of the Smo receptor as the bait. Several positive clones were identified to encode the C-terminal portion of Miz1. To confirm the interaction between full-length Smo and Miz1, Flag-tagged Smo (Flag-Smo) was co-transfected with Myc-tagged Miz1 (Myc-Miz1) or a POZ domain deletion mutant Miz1 (Myc-Miz1ΔPOZ) into HEK293 cells. Both the full-length and ΔPOZ Miz1 co-immunoprecipitated with Smo (Figure 1A and 1B), indicating that Miz1 interacts with Smo, and the POZ domain of Miz1 is not required for such interaction.

To further investigate how Miz1 is involved in the activation of Hh signaling, we performed co-immunoprecipitaion experiments to determine if Miz1 binds Gli2. Indeed, the interaction between Flag-tagged Miz1 (Flag-Miz1) and HA-tagged Gli2 (HA-Gli2) was detected in transfected HEK 293 cells (Figure 1C). Interestingly, deletion of the POZ domain in Miz1 completely abrogated the binding between Miz1 and Gli2 (Figure 1A and 1C), suggesting that Miz1 has the potential to serve as a scaffolding protein by utilizing different domains to bring Smo and Gli2 into a signaling complex.

We next determined the effect of Miz1 on Hh signaling by monitoring Gli-luciferase reporter activity in the presence of Miz1 overexpression using pcDNA3 as a negative control and Smo overexpression as a positive control in C3H10T1/2 cells. Miz1 activated the reporter to the same extent as Smo, 5.73±0.09 and 4.30±0.76 fold respectively. Deletion of the POZ domain of Miz1 completely abolished the activation (1.18±0.13, Figure 1A and 1D). Additionally, deletion of the Myc binding domain (amino acids 641–715) in Miz1 [17] did not affect its ability to activate Gli signaling (5.99±0.53, Figure 1A and 1D), indicating that the interaction between Myc and Miz1 is not required for its ability to upregulate Gli activity. Furthermore, to investigate whether Miz1-induced activation of Gli depends on Smo, the effect of Miz1 on the transcriptional activity of Gli was determined in Smo knockout (*Smo^−/−^*) MEF cells. Transient expression of Smo elevated reporter activity relative to control (pcDNA3 1.00±0.10; Smo 3.93±0.21, Figure 1F). Interestingly, expression of Miz1 alone activated the Gli reporter to the same extent as overexpressing Smo, suggesting that the contribution of Miz1 to Gli signaling occurs downstream of Smo activation. Consistent with the result that the POZ domain is required for binding Gli2, overexpression of the POZ domain deletion of mutant Miz1 did not change Gli reporter activity (Miz1ΔPOZ 1.20±0.10, Figure 1A and 1F). Taken together, our findings identified Miz1 as an activator of Hh signaling by promoting signaling relay from Smo to Gli.

### Silencing endogenous Miz1 inhibits Hh Signaling

**Figure 2:**
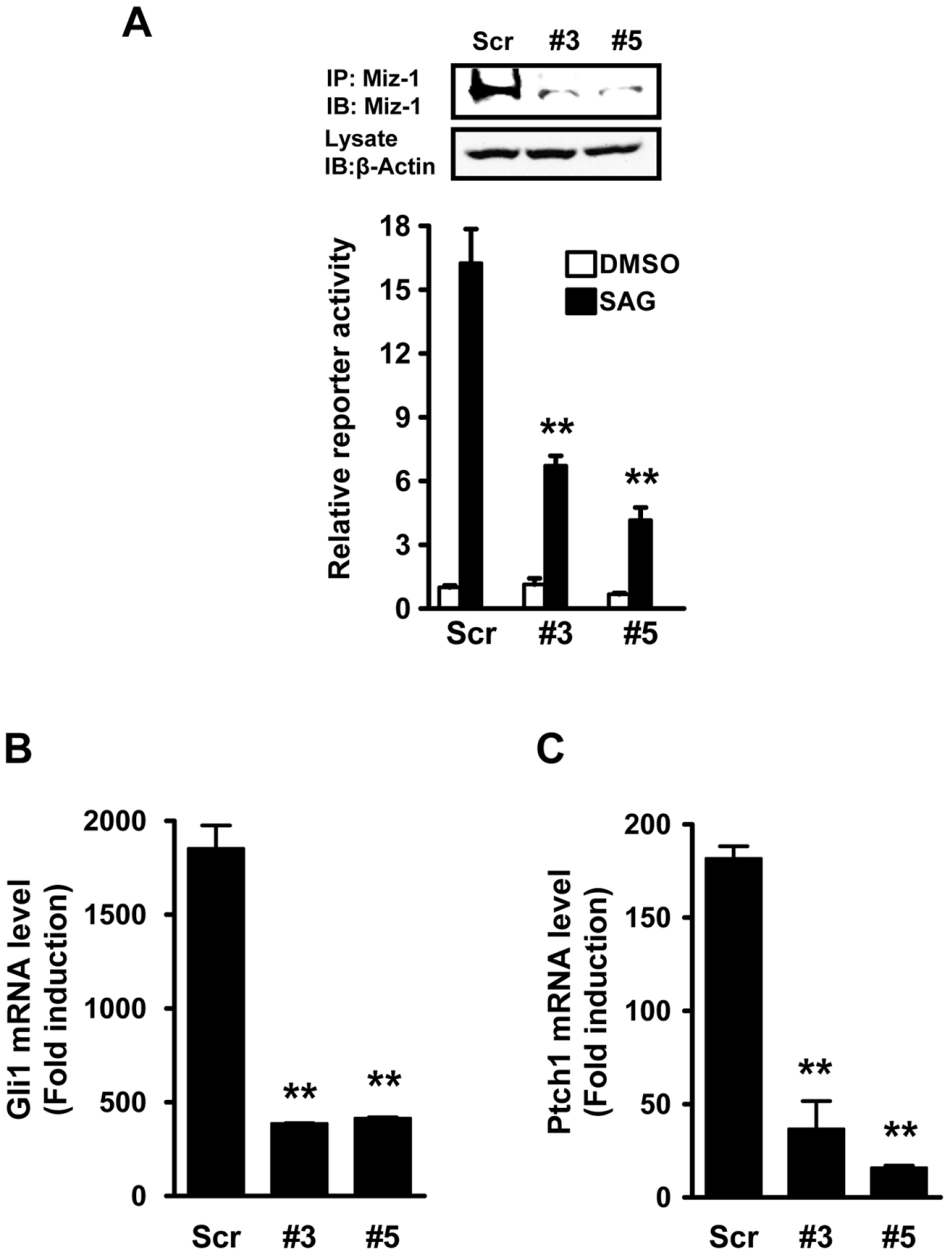
Knockdown of endogenous Miz1 attenuates Hh signaling. (A) Knock-down of Miz1 diminishes SAG-dependent activation of Gli activity. Stable NIH 3T3 cells infected with either a scrambled shRNA (Scr) or Miz1 shRNA lentiviruses (#3 and #5) were transfected with the Hh-responsive reporter and treated with DMSO or SAG. The Gli reporter activity was determined using the ratio of firefly/*Renilla* luciferase light-units. The value in Scr control cells treated with DMSO was set to 1, and relative Gli activities for all other conditions was normalized accordingly. Data shown in the graph represent the mean ± SEM (n = 3, **: *p*<0.001, compared to the SAG-stimulated Scr sample, t test). The knockdown efficiency of Miz1 was verified and shown in upper panels. Specifically, endogenous Miz1 was immunoprecipitated from cell lysates and subsequently detected by immunoblotting using the Miz1 antibody. β-actin was used as a loading control. (B) and (C) Loss of Miz1 decreases the mRNA expression of Hh target genes. The scrambled control (Scr) and Miz1 knockdown (#3 and #5) cells were treated with DMSO or SAG for 24 hours. The mRNA levels of Gli1 (B) and Ptc1 (C) were analyzed using qRT-PCR. Data shown in both graphs represent the mean ± SEM (n = 3, **, *p*<0.001, compared to the Scr sample, t test).

To determine the role of endogenous Miz1 in regulating Hh signaling, we utilized lentivius-based shRNA to knockdown Miz1 levels and measured Gli luciferase reporter activity in the presence of a potent Smo agonist, SAG, in NIH 3T3 cells. We identified two shRNA targeting sequences (Miz1 shRNA #3 and shRNA #5, and indicated as #3 and #5, respectively) specific for knocking-down endogenous Miz1 (Figure 2A). In control cells exposed to scrambled shRNA, SAG activated the Gli reporter 16.2±1.6 fold. In each of the Miz1 knockdown cell lines, SAG-induced activation was substantially decreased (6.72±0.46 and 4.16±0.60 in #3 and #5 respectively, Figure 2A), indicating that Miz1 is necessary for the optimal activation of Hh signaling. Furthermore, since the activation of the Hh pathway leads to elevated Gli1 and Ptch1 mRNA expression, we assessed changes in mRNA level of these Hh target genes in response to Miz1 depletion. Control and Miz1 knockdown cells were treated with DMSO or SAG. In scrambled control cells, SAG treatment greatly increased Gli1 and Ptch1 mRNA levels by 1854.0±121.5 and 181.7±6.5 fold, respectively, whereas the level of Gli1 and Ptch1 mRNA induction was significantly inhibited in Miz1 knockdown cells (Figure 2B, Gli1 mRNA induction fold, #3, 386.3±1.7 and #5, 414.6±5.7, and Figure 2C, Ptch1 mRNA induction fold, #3, 36.7±15.0 and #5, 15.8±1.2). Collectively, these results confirmed that Miz1 plays a positive role in controlling Hh signaling.

### Miz1 traffics into primary cilia upon Smo activation

**Figure 3:**
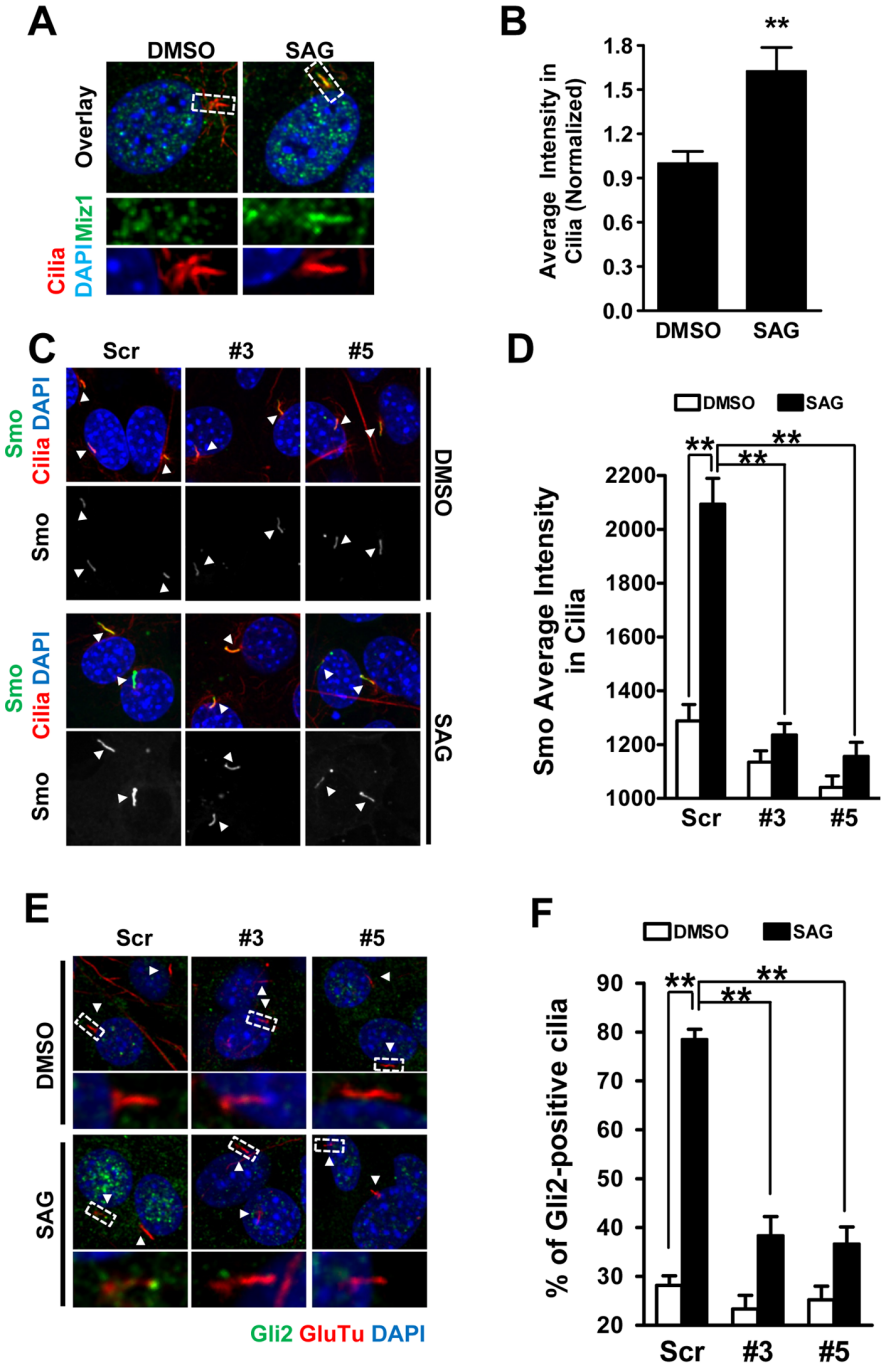
Miz1 regulates Hh signaling in primary cilia. (A) Confocal images showing the localization of Miz1 in primary cilia. NIH 3T3 cells treated with DMSO or SAG were co-stained with antibodies for Miz1 (green color) and acetylated-tubulin (red color, to indicate primary cilia). Cell nuclei were stained with DAPI (blue color). Co-localization is shown in the overlay panel. Middle and bottom panels correspond to the regions in the dashed white boxes in the upper panels. (B) Quantification of the average intensity of Miz1 in primary cilia. Total 101 and 103 cilia were quantified for DMSO and SAG treated group, respectively (**, *p*<0.001, compared to the DMSO sample, t test). (C) Knockdown of Miz1 expression inhibits activation-dependent recruitment of Smo into primary cilia. NIH 3T3 cells expressing GFP-Smo were stably infected with scrambled (Scr) control or Miz1 shRNAs (#3 and #5). Cells treated with DMSO or SAG were stained with anti-acetylated tubulin (red color, to indicate primary cilia), and cell nuclei were indicated by DAPI staining (blue color). GFP-Smo was shown in green color. Overlay images are shown in upper panels while the single-channel GFP-Smo images are shown directly below. Arrowheads in each image indicate primary cilia. (D) Quantification of the average intensity of GFP-Smo in primary cilia. Data shown in the graph represent the mean ± SEM (n = 37, 204, 267, 268, 215, and 212 cilia from left to right; **: *p*<0.001, t test). (E) Knockdown of Miz1 inhibits the accumulation of Gli2 at the tip of primary cilia upon Hh activation. NIH 3T3 cells were stably infected with lentiviral particles encoding either scrambled control shRNA (Scr) or shRNAs against Miz1 (#3 and #5). Cells were treated with DMSO or SAG for 24 hours and subsequently co-stained with antibodies for Gli2 (green color) and detyrosinated-tubulin (GluTu, red color, to indicate primary cilia). Cell nuclei were stained with DAPI (blue color). Overlay images are shown in upper panels for each condition, and the enlarged images of areas enclosed by the dashed white boxes are shown in the panels directly below. (F) Quantification of the percentage Gli2-containing primary cilia. Data shown in the graph represent the mean ± SEM (n = 276, 406, 214, 239, 246, and 307 cilia from left to right. **: *p*<0.001, t test).

The primary cilium plays a critical role in Hh signaling pathway [7]. Many Hh key components have been found to localize in primary cilium upon Hh activation [10], [11], [12], [13], [14], [15]. To determine whether Miz1 is involved in primary cilium-dependent Hh activation, we first examined the subcellular localization of endogenous Miz1 in primary cilia by confocal microscopy in NIH3T3 cells. Cells treated with either DMSO or 0.25 μM SAG were stained with the anti-Miz1 antibody and an antibody for the primary cilium marker acetylated tubulin. A significant increase of Miz1 was observed in primary cilia after SAG stimulation (DMSO 1.00±0.08, SAG 1.65±0.16) (Figure 3A and 3B). To verify whether the transport of Miz1 into primary cilia depends on Smo activity, we performed the same experiment on *Smo^−/−^* MEF. We did not observe any ciliary localization of Miz1 in *Smo^−/−^* MEF (DMSO, 1.00±0.16 and SAG, 1.18±0.19). These results indicate that Smo activation is essential for the trafficking of Miz1 into cilia.

### Miz1 regulates Smo trafficking into primary cilia in an activity dependent manner

We further studied whether Miz1 is involved in the regulation of Smo trafficking into the primary cilia upon stimulation. Due to poor detection of endogenous Smo using commercially available antibodies, we performed the experiments in NIH3T3 cells stably expressing GFP-tagged Smo (GFP-Smo) [10]. Consistent with previous findings [10], GFP-Smo is constitutively active and accumulates in primary cilia even without stimulation (Figure 3C). To test whether Miz1 is required for the recruitment of GFP-Smo to cilia, the localization patterns of GFP-Smo were analyzed in scrambled control and Miz1 knockdown NIH 3T3 cells. Knockdown of Miz1 had no effect on ciliogenesis as primary cilia were detected in similar numbers of control and knockdown cells (Scr, 66%±3%; #3, 68%±3%; and #5, 60%±3%), and no changes in cilium length and width were observed. In the absence of stimulation, a slight but significant decrease of GFP-Smo accumulation in primary cilia was observed in Miz1 knockdown cells (Figure 3C and 3D, Scr, 1288±60; #3, 1135±42; and #5, 1041±43). SAG stimulation triggered further recruitment of GFP-Smo into primary cilia in control cells (Scr, 2094±96), however, this increase was completely inhibited in Miz1 knockdown cells (#3, 1236±42 and #5, 1156±52) (Figure 3C and 3D). Collectively, these results suggest that the recruitment of Smo and Miz1 into primary cilia requires the formation of a Smo-Miz1 complex as loss of either protein impairs the translocation process.

### Miz1 facilitates Gli2 recruitment to the tip of primary cilia

Furthermore, to test whether Miz1 is involved in the accumulation of Gli2 at the tip of primary cilia upon Hh activation, we stained Gli2 with the anti-Gli2C 1H6 antibody, which detects only the full length of Gli2 [12]. Consistent with the previous study [12], Gli2 accumulated at the distal tips of cilia in up to 80% of cells following SAG stimulation (in Scr DMSO 28.16±1.97%, SAG 78.54±2.02%) (Figure 3E and 3F). Knockdown of Miz1 greatly decreased the percentage of Gli2-containing cilia upon SAG activation (#3 DMSO 23.34±2.81%, SAG 38.40±3.83%; #5 DMSO 25.20±2.80%, SAG 36.67±3.44%) (Figure 3E and 3F). Thus, the accumulation of Gli2 at the tip of primary cilia is facilitated by binding to Miz1.

### Miz1 translocates into the nucleus upon Smo activation

**Figure 4:**
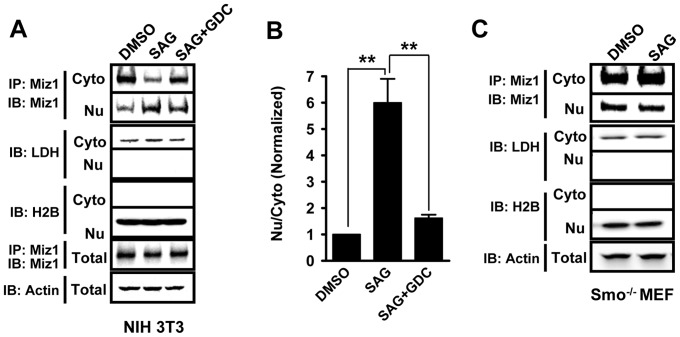
Miz1 is transported into nucleus upon Hh activation. (A) NIH 3T3 cells treated with DMSO, 0.25 μM SAG, or 0.25 μM SAG plus 1 μM GDC-0449 (SAG+GDC) were fractionated into cytosol (Cyto) and nuclear (Nu) fractions. Endogenous Miz1 was immunoprecipitated from each fraction first and analyzed using immunoblotting. The presence of Miz1 was detected using the Miz1 antibody. Lactate dehydrogenase (LDH) and Histone H2B (H2B) were used as protein markers of cytosol and nuclear fractions, respectively. β-actin was used as the loading control. (B) Graphic representation of data shown in panel (A). The amount of Miz1 detected in the nuclear fraction was normalized to that of cytosol, and this number (Nu/Cyto) for the DMSO treated group was set to 1. Data shown in the graph represent the mean ± SEM (n = 3; **: *p*<0.001, t test). (C) Smo is required for Miz1 translocation into the nucleus. *Smo^−/−^* MEF cells treated with DMSO or SAG were fractionated into cytosol (Cyto) and nuclear (Nu) fractions. Endogenous Miz1 was immunoprecipitated from each fraction first and analyzed using immunoblotting. Note that the amount of Miz1 in the cytosol or nucleus is not changed upon SAG treatment in *Smo^−/−^* MEF.

Miz1 does not possess a nuclear localization signal, but Miz1 can locate in both cytosol and nucleus [17]. To study the distribution of Miz1 upon Hh activation, NIH 3T3 cells were fractionated into cytoslic and nuclear components, and lactate dehydrogenase (LDH) and Histone 2B (H2B) were used as markers for cytosol and nucleus, respectively. Activation of Smo resulted in a 6-fold increase in the amount of Miz1 accumulated in the nuclear fraction in SAG treated cells, and this accumulation was greatly blocked by the Smo antagonist GDC-0449 (Figure 4A and 4B, SAG, 6.00±0.91 and SAG+GDC-0449, 1.62±0.13). The total Miz1 protein level was not changed by the treatment, indicating that the increase of Miz1 in the nuclear fraction is not due to an increase of Miz1 expression. In contrast, the amount of Miz1 in the nuclear fraction was not altered by the SAG treatment in *Smo^−/−^* MEF cells, suggesting that the nuclear translocation of Miz1 is a Smo activation-dependent process (Figure 4C).

### Knockdown of Miz1 inhibits Gli2 trafficking into the nucleus upon Hh activation

**Figure 5:**
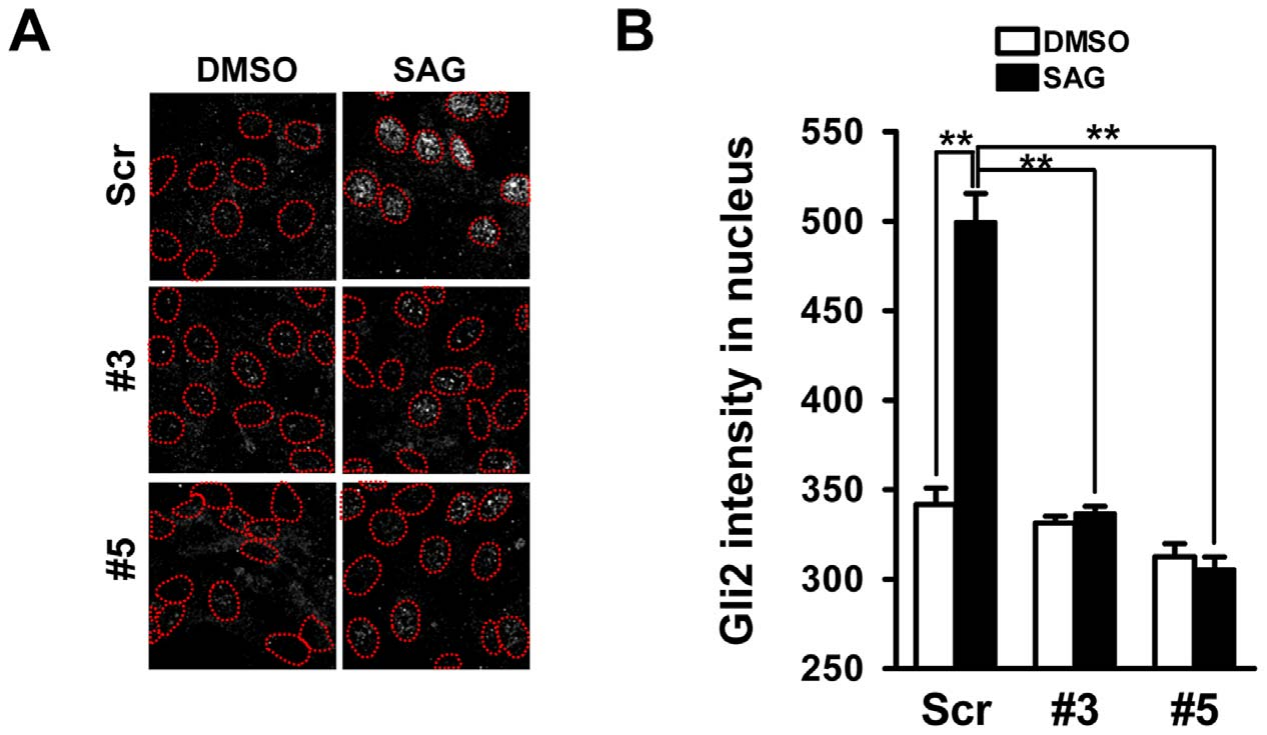
Miz1 is required for nuclear translocation of Gli2. (A) NIH 3T3 cells were stably infected with lentivirus encoding either the scrambled control shRNA (Scr) or shRNAs against Miz1 (#3 and #5). Cells treated with DMSO or 0.25 μM SAG for 24 hours were stained with the Gli2 antibody. Cell nuclei were stained with DAPI. Red dashed circles adapted from corresponding DAPI images indicate the regions of nuclei. (B) Graphic representation of data shown in panel (A). The amount of Gli2 in the nucleus was measured as the intensity of Gli2 fluorescence staining within the DAPI-positive nuclear regions [circled areas in images shown in (A)]. Data represent the mean ± SEM (n = 203, 127, 211, 210, 200 and 241 cells from left to right, respectively. **: p<0.001, t test).

Studies have shown that the Gli2 accumulation at the tip of primary cilia within minutes of Hh stimulation is followed by its trafficking to the nucleus [12], [13]. However, it is not clear how this process is regulated. We demonstrated above that Miz1 is translocated to primary cilia (Figure 3) and subsequently accumulated in the nucleus upon activation of Smo (Figure 4). To examine whether Miz1 facilitates Gli2 nuclear translocation, we quantified the intensity of Gli2 in the nucleus by immunofluorescence staining in the presence or absence of SAG treatment (Figure 5A). Approximately a 1.5 fold increase of Gli2 nuclear localization was observed after SAG stimulation in scrambled control cells (in Scr, DMSO, 341.8±9.0, and SAG, 499.5±15.9), whereas knockdown of Miz1 completely prevented Gli2 nuclear recruitment (Figure 5B, in #3, DMSO, 331.3±3.9 and SAG, 336.8±3.9; and in #5, DMSO, 312.5±7.4 and SAG, 305.4±7.0). These results indicate that Miz1 regulates Hh signaling by facilitating the nuclear recruitment of Gli2.

### Loss of Miz1 in medulloblastoma cells suppresses cell proliferation and tumorigenesis

**Figure 6:**
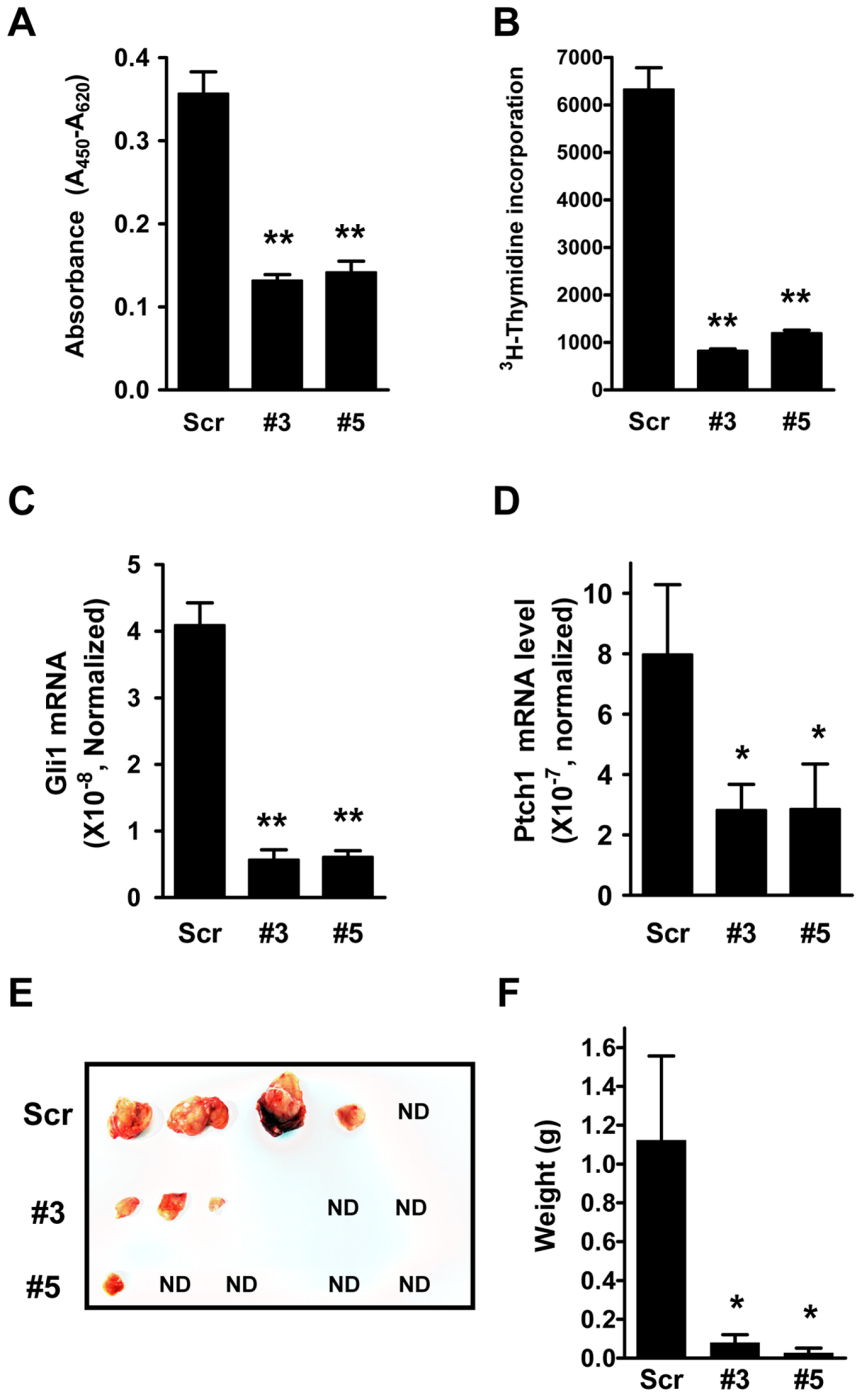
Knockdown of Miz1 inhibits cell proliferation and tumorigenesis in PZp53^MED1^ medulloblastoma cells. (A) and (B) The rate of cell proliferation was measured in stable scrambled control (Scr) and Miz1 knockdown (#3 and #5) PZp53^MED1^ cells using WST-1 (A) and ^3^H-thymidine incorporation assays (B). Data shown in the graphs represent the mean ± SEM (n = 3; **: *p*<0.001, compared to the Scr sample, t test). (C) and (D) The levels of mRNA expression for Gli1 (C) and Ptch1 (D) were measured using qRT-PCR in control and Miz1 knockdown PZp53^MED1^ cells. Data shown in the graphs represent the mean ± SEM (n = 4 for Gli1 and n = 3 for Ptch1; *: *p*<0.05; and **: *p*<0.001, compared to the Scr sample, t test). (E) SCID mice were inoculated subcutaneously with stable control and Miz1 knockdown PZp53^MED1^ cells. Image shows the tumors dissected from mice at the end of the experiment. ND represents non-detectable tumor growth around the injection sites. (F) Quantitative representation of tumor weights measured. Data in the graph represent the mean ± SEM (n = 5 for each group; *: *p*<0.05, compared to the Scr sample, t test).

Aberrant activation of Hh signaling has been implicated in many types of cancer [3], [4], [5], [6]. Mutations in Ptch1 and Smo occur in patients with medulloblastoma (MB), an aggressive childhood cancer arising from cerebellar GNPs [27]. To test the function of Miz1, we stably knocked down the endogenous expression of Miz1 in PZp53^MED1^ cells generated from a murine Hh-driven medulloblastoma allograft model [24]. Loss of Miz1 significantly reduced cell proliferation as assessed using WST-1 assays (Figure 6A, Scr, 0.35±0.03; versus #3, 0.13±0.01 and #5, 0.14±0.01) and ^3^H-thymidine incorporation assays (Figure 6B, Scr, 6326±457; versus #3, 824±40 and #5, 1193±64). The suppression of cell growth coincides with a reduction in the mRNA levels of Hh target genes Gli1 and Ptch1 (Figure 6C and 6D, for Gli1 mRNA, Scr, 4.09±0.32, versus #3, 0.57±0.15 and #5, 0.61±0.09; and for Ptch1 mRNA, Scr, 7.97±2.31, versus #3, 2.82±0.86 and #5, 2.85±1.49), suggesting decreased Hh signaling is responsible for the growth inhibition observed in Miz1 knockdown cells.

Furthermore, we examined the tumor growth of Miz1 knockdown PZp53^MED1^ cells *in vivo* in SCID mice. Control and Miz1 knockdown cells were mixed with Matrigel and injected subcutaneously into SCID mice. The growth of tumors was monitored daily until the largest tumor reached 2000 mm^3^. At this time point, all tumors were harvested and weighed. In the scrambled control group, tumors were observed in 4 out of 5 mice (Scr, 0.31 g, 1.37 g, 2.40 g, and 1.53 g), and only one mouse had no detectable tumor growth (Figure 6E and 6F). In marked contrast, tumor formation was significantly inhibited in the Miz1 knockdown group and the incidence was dramatically decreased. Specifically, in the #3 Miz1 knockdown group, 2 out 5 mice were without detectable tumors; and in the #5 group, 4 out of 5 mice had none (Figure 4E–F, #3, 0.11 g, 0.05 g and 0.23 g; and #5, 0.13 g). Taken together, our results indicate that Miz1 functions as a potential oncogene in medulloblastoma by promoting the signal transduction of the Hh pathway.

## Discussion

In the present study, we have demonstrated that Miz1 is a novel positive regulator in the Hh signaling pathway. Miz1 can bind to both the Smo receptor and downstream Gli2 and regulate the localization of Gli2 in the primary cilium upon activation. In addition, Miz1 also facilitates the translocation of Gli2 into the nucleus. More significantly, knockdown of Miz1 in Hh-driven medulloblastoma cells arrests tumor growth *in vitro* and *in vivo*.

We have shown Miz1 interacts with Smo receptor and Gli2. However we don't know whether Miz1 binds to activated Smo or inactivated Smo. Further detailed studies need to be done to address this question. We have also investigated whether Gli1 and Gli3 may also interact with Miz1. However no obvious interaction between Gli1 and Miz1 or Gli3 and Miz1 was observed.

Hh signaling pathway was first identified in a drosophila genetic screen for mutants affecting body patterning in the early 1980s [28]. More proteins have been found involved in regulating the Hh pathway since then [26]. Here, we have identified Miz1 as an important activator in Hh signaling. Miz1 itself is a transcription factor and can regulate the transcription of several cell cycle regulatory proteins such as p15Ink4b and p21Cip1 [19], [20], [21], [29]. Previous studies have shown that Miz1 interacts with Myc, and this interaction is required for repressing the transcriptional activity of Miz1 [17]. We demonstrate here that deletion of the Myc-interacting domain in Miz1 (Miz1Δ641-715) has no effect on Miz1-dependent activation of Gli reporter activity (Figure 1A and 1B), suggesting that Myc and Miz1 interaction is not involved in regulating Hh signaling. Meanwhile, our results indicate that Miz1 facilitates the translocation of the transcription factor Gli2 into the nucleus and triggers the activation of Hh downstream signaling. However, further studies are needed to determine whether the transcriptional activity of Miz1 is involved in the regulation of Hh signaling.

It has been suggested that Hh signaling is initiated by activating Smo in primary cilia that leads to the recruitment of Gli into cilia and the subsequent activation [14], [30]. Specifically, activation of Smo results in the dissociation of SuFu from Gli2, thus allowing Gli2 to translocate into the nucleus and become active [14], and this entire process depends on the integrity of the primary cilium [14], [30]. In our study, we show that Miz1 is not involved in ciliogenesis. However, Miz1 is localized to primary cilia upon Smo activation (Figure 3A–B). Miz1 also regulates the trafficking of Smo into primary cilia in an activity dependent manner (Figure 3C–D). Unlike Sufu and Gli2 concentrating only at the tip of the cilia upon Hh activation, the distribution of Miz1 in cilia co-localizes with acetylated tubulin, a primary cilia marker, similar with the pattern of Smo in cilia. These data indicate that Smo and Miz1 form a protein complex and thus regulate downstream signaling. The recruitment of Miz1 in cilia can further enrich Gli2 at the tip of cilium and subsequently both Miz1 and Gli2 translocate into nucleus, indicating that Miz1 facilitates the trafficking of Gli2 into nucleus as well.

Furthermore, we find that Miz1 plays an important role in promoting cell proliferation and tumorigenicity in Hh-driven medulloblastoma cells. Previously, it has been shown that Miz1 acts as a negative regulator of cell proliferation because it induces the expression of cell-cycle inhibitors such as p21Cip1 and p15Ink4 [19], [20], [21], [29]. However, *Miz1^−/−^* embryos show impaired cell proliferation and failed to survive beyond E6.5 [22]. In addition, studies from Ziegelbauer et al. and others have suggested that Miz1 can promote cell survival and growth by suppressing apoptosis pathways [31], [32], [33], [34]. We find here that loss of Miz1 expression causes cell and tumor growth arrest in medulloblastoma by down-regulation of Hh signaling. These cellular and *in vivo* studies provide the first evidence that Miz1 promotes the tumorigenesis of medulloblastoma by enhancing Gli-mediated Hh signaling, thus supporting a potential oncogenic role of Miz1. Further understanding of the functional contribution of Miz1 in tumor progression will help to develop new therapeutic strategies in targeting Hh-driven cancers.

In summary, these studies provide the first evidence that the transcription factor Miz1 regulates the Hh signaling pathway, and plays a positive role in Hh driven proliferation. Miz1 is a potential therapeutic target in Myc-driven cancers. However, strategies to modulate Miz1 will need to consider how they also impact Hh signaling.
